# A simple, rapid, and reliable protocol to localize hydrogen peroxide in large plant organs by DAB-mediated tissue printing

**DOI:** 10.3389/fpls.2014.00745

**Published:** 2014-12-22

**Authors:** Yong-Hua Liu, Christina E. Offler, Yong-Ling Ruan

**Affiliations:** ^1^Australia–China Research Centre for Crop Improvement and School of Environmental and Life Sciences, The University of NewcastleCallaghan, NSW, Australia; ^2^Institute of Vegetables, Zhejiang Academy of Agricultural SciencesHangzhou, China

**Keywords:** fruits and stems, hydrogen peroxide, localization, tissue printing, reactive oxygen species

## Abstract

Hydrogen peroxide (H_2_O_2_) is a major reactive oxygen species (ROS) and plays diverse roles in plant development and stress responses. However, its localization in large and thick plant organs (e.g., stem, roots, and fruits), other than leaves, has proven to be challenging due to the difficulties for the commonly used H_2_O_2_-specific chemicals, such as 3,3′-diaminobenzidine (DAB), cerium chloride (CeCl_3_), and 2′,7′-dichlorofluorescin diacetate (H_2_DCF-DA), to penetrate those organs. Theoretically, the reaction of endogenous H_2_O_2_ with these chemicals could be facilitated by using thin organ sections. However, the rapid production of wound-induced H_2_O_2_ associated with this procedure inevitably disturbs the original distribution of H_2_O_2_ *in vivo*. Here, by employing tomato seedling stems and fruits as testing materials, we report a novel, simple, and rapid protocol to localize H_2_O_2_ in those organs using DAB-mediated tissue printing. The rapidity of the protocol (within 15 s) completely avoided the interference of wound-induced H_2_O_2_ during experimentation. Moreover, the H_2_O_2_ signal on the printing was stable for at least 1 h with no or little background produced. We conclude that DAB-mediated tissue printing developed here provide a new feasible and reliable method to localize H_2_O_2_ in large plant organs, hence should have broad applications in studying ROS biology.

## INTRODUCTION

Reactive oxygen species (ROS)accumulate when plants are under various biotic (pathogen attack) and abiotic (e.g., high light, drought, heat, salt, and heavy metal) stresses (Apel and Hirt, 2004; Suzuki et al., 2012; Choudhury et al., 2013). On one hand, excessive ROS cause oxidative damage to proteins, DNA, and lipids. On the other hand, ROS also act as signaling molecules to regulate development and stress responses (Apel and Hirt, 2004). There are different kinds of ROS in plants, including singlet oxygen(^1^O_2_), superoxide ($O_{2}^{-}$), H_2_O_2_, and hydroxyl radical (OH^-^). Among them, H_2_O_2_ is thought to be relatively stable (Bienert et al., 2007) and the most likely signaling ROS to regulate developmental and stress responses (Van Breusegem et al., 2008), and thus one of the most studied ROS species.

Hydrogen peroxide could be detected quantitatively and qualitatively. Accurate quantification of H_2_O_2_ in plant organs, however, is difficult to achieve owing to the unique properties of H_2_O_2_ being highly metabolically active with a half-life of only 1 ms in plants (Reth, 2002; Veljovic-Jovanovic et al., 2002; Petrov and Van Breusegem, 2012). Even storage of plant materials at -80°C may result in the loss of H_2_O_2_ by as much as 60% within 7 days (Cheeseman, 2006). Moreover, H_2_O_2_ may react with many reduced compounds released during homogenization of plant materials, such as ascorbic acid which leads to underestimation of H_2_O_2_ levels. The level of H_2_O_2_ could also be overestimated because of endogenous phenolics in plant tissues (Veljovic-Jovanovic et al., 2002). In fact, it has been reported that H_2_O_2_ content can span more than several orders of magnitude even for leaves from the same species (from nM to mM; Cheeseman, 2006; Razem, 2008), indicating major challenges in H_2_O_2_ quantification.

Hydrogen peroxide can also be qualitatively localized at a tissue or cellular level. Compared to the measurement of H_2_O_2_ extracted from whole plant organs, this approach has the advantage of localizing H_2_O_2_ in particular cellular sites in a multicellular tissue or organ, thereby potentially providing deep insights into the cellular origin and function of the H_2_O_2_. Localization of H_2_O_2_ relies on histochemical staining of plant organs. The most commonly used chemicals to localize H_2_O_2_ *in planta* are 3,3′-diaminobenzidine (DAB), cerium chloride (CeCl_3_), and 2′,7′-dichlorofluorescin diacetate (H_2_DCF-DA). However, detection of H_2_O_2_ with DAB requires a long incubation time with DAB solution. For example, H_2_O_2_ localization in detached leaves and tender seedling roots usually needs more than 8 h incubation in DAB solution (Thordal-Christensen et al., 1997; Salzer et al., 1999). To gain higher resolution of the cellular localization of H_2_O_2_, a TEM method has also been employed. In this method, endogenous H_2_O_2_ reacts with exogenously supplied CeCl_3_ to form cerium perhydroxide, which gives dark deposits under TEM. However, before samples are fixed for TEM, leaves must be incubated in CeCl_3_ solution for at least 1 h to allow the penetration of CeCl_3_ into the tissue and the formation of cerium perhydroxide (Bestwick et al., 1997; Romero-Puertas et al., 2004). Such a long period of incubation of detached plant organs in DAB and CeCl_3_ solution would inevitably change the distribution pattern of H_2_O_2_ *in vivo*, because of both the rapid degradation of original H_2_O_2_ due to its short half-life and the *de novo* production of wound-induced H_2_O_2_. Queval et al. (2008) has suggested that DAB staining only reflect the production of H_2_O_2_ rather than its original concentration or distribution. Indeed, it has been reported that H_2_O_2_ accumulates rapidly at the cutting site of *Arabidopsis* stem, within 1 min after cutting, and furthermore the wound-induced H_2_O_2_ signal can travel at a speed of 8.4 cm min^-1^ and induce dramatic increase of H_2_O_2_ in distal cotyledons within 2 min (Miller et al., 2009).

Although H_2_DCF-DA staining takes a shorter incubation time (10 min) than DAB and CeCl_3_ staining, it is mostly used for the visualization of H_2_O_2_ on the surface of plant organs such as, epidermis of citrus fruit (Macarisin et al., 2007) and tobacco leaves (Essmann et al., 2008). To the best of our knowledge, there have been no reports on using the above-mentioned three chemicals to localize H_2_O_2_ in the inner parts of large plant organs (e.g., stem and fruit). The most likely reason for this scenario is the difficulties for these chemicals to infiltrate into the large plant organs. This was indeed the case in our preliminary studies on tomato ovaries using DAB and H_2_DCF-DA staining. Only recently, H_2_O_2_ localization in seed has been reported in a study on rice using H_2_DCF-DA (Nagasawa et al., 2013), in which seeds were sectioned in half before staining. Although sectioning facilitated the reaction of H_2_DCF-DA with H_2_O_2_, the rapid production of wound-induced H_2_O_2_ could dramatically alter the original distribution of H_2_O_2_ and yield an artifact of overestimate of H_2_O_2_ level, just as discussed above. Thus, how to avoid or minimize the interference of wound-induced H_2_O_2_ is a prerequisite for the accurate localization of H_2_O_2_ in organ sections.

Some studies have endeavored to tackle the problem of wound-induced production of H_2_O_2_. For example, starch/KI-mediated tissue printing has been used to localize H_2_O_2_ in the stem of seedlings from different plant species including soybean, pea, common bean, sunflower, and cucumber (Schopfer, 1994). In this procedure, the sections of seedling stem were pressed for 60 s, immediately after cutting, on nitrocellulose paper impregnated with starch/KI solution. The oxidation of KI to I_2_ by H_2_O_2_ can produce the blue–black I_2_-starch complex (Olson and Varner, 1993), which can be photographed under the microscope. The whole procedure can be completed in just 70 s, and thus wound-induced H_2_O_2_ can be avoided to a large extent (Schopfer, 1994). However, the intensity of color increases with time after printing, and therefore the signal must be observed and recorded immediately (Schopfer, 1994). In addition, there is a background color due to continuous autoxidation of KI, which interferes and blurs the results (Neves et al., 1998; Przymusiński et al., 2007).

After being absorbed into plant cells, DAB reacts with H_2_O_2_ to form a reddish-brown polymer in the presence of peroxidase (Thordal-Christensen et al., 1997). DAB-mediated tissue printing has been employed to localize peroxidase in plants (Spruce et al., 1987). However, there has been no report on DAB-mediated tissue printing to directly localize H_2_O_2_ in plants. Here, we described such a novel procedure. The new protocol can rapidly and reliably localize H_2_O_2_ in sections of large tomato organs, namely stems and fruits. The whole procedure was completed within 15 s which avoided the interference of wound-induced H_2_O_2_. At the same time, the signal was very stable for at least 1 h with little background produced.

## MATERIALS AND METHODS

### H_2_O_2_ LOCALIZATION BY DAB-MEDIATED TISSUE PRINTING

Nitrocellulose membrane (0.45 μm in pore size, Hybond^TM^-C Extra, Amersham) was soaked in 5 mg mL^-1^ DAB–HCl solution (pH 3.8) and then air-dried at room temperature for 30 min in the dark. The soaked nitrocellulose membrane was placed on a layer of un-soaked nitrocellulose membrane, which can absorb excessive plant exudate from cutting site during tissue printing. Tissue printing was performed at ∼20°C. Free-hand sections in 1.0 mm thickness were prepared with a razor blade. The sections were cut from the top, middle, and bottom positions of stems of 50-days old seedlings, or transversely from the middle of fruits at 5 and 10 DAF. The sections were gently pressed onto the impregnated nitrocellulose membrane with forefinger for 10 s to ensure that H_2_O_2_ in sections is successfully transferred to membrane and at the same time the sections are not crushed by the press. Then, the sections were carefully removed with forceps. This, together with the 5 s required for the cutting of the section, renders the total time for tissue printing being only 15 s. The membrane was then washed in 100% ethanol to remove the possible interfering substance (e.g., chlorophyll) and photographed under a dissection microscope after 5 min at room temperature to allow completion of the reaction between the H_2_O_2_ derived from plant cells and DAB pre-soaked in the membrane.

To verify the specificity of reaction, tissue printings were also done as above on membranes pre-soaked in 5 mg mL^-1^ DAB–HCl solution (pH 3.8) containing 100 mM ascorbic acid. To test whether there is a production of wound-induced H_2_O_2_ under our experimental conditions, tissue printing was performed at 0, 1, and 2 min after sectioning. To test if endogenous peroxidase was sufficient to support the reaction, H_2_O_2_ was introduced exogenously by soaking nitrocellulose membrane in 5 mg mL^-1^ DAB–HCl solution (pH 3.8) containing 20 mM H_2_O_2_ and tissue printing was done as described above. If the endogenous peroxidase is sufficient, it can be expected that the tissue printing with exogenously supplied H_2_O_2_ would produce stronger signals.

### H_2_O_2_ QUANTIFICATION IN TOMATO STEMS

Hydrogen peroxide extraction was carried out according to Veljovic-Jovanovic et al. (2002). Briefly, 100 mg of stem from the top, middle, and bottom part of tomato seedlings was harvested, snap-frozen in liquid nitrogen and analyzed immediately. Samples were homogenized in 1.5 mL 1 M HClO_4_ with 100 mg of insoluble polyvinylpyrrolidone, which can remove phenolic compounds. Homogenates were centrifuged at 13000 × *g* for 10 min at 4°C. The H_2_O_2_ content in the supernatant was then determined as described by Cheeseman (2006). Briefly, 60 μL extract was mixed with 600 μL eFOX reagents (containing 250 μM ferrous ammonium sulfate, 100 μM sorbitol, 100 μM xylenol orange, and 1% ethanol in 25 mM H_2_SO_4_). Then, the difference in absorbance between 550 and 800 nm was recorded at least 30 min after mixing the supernatant with the eFOX reagents. The content of H_2_O_2_ was calculated using a standard curve of H_2_O_2_.

### STATISTICAL ANALYSIS

One-way ANOVA was done using IBM SPSS Statistics 20.

## RESULTS AND DISCUSSION

### THE DAB-MEDIATED TISSUE PRINTING FOR LOCALIZING H_2_O_2_ IS SIMPLE, RAPID, AND RELIABLE

**FIGURE 1:**
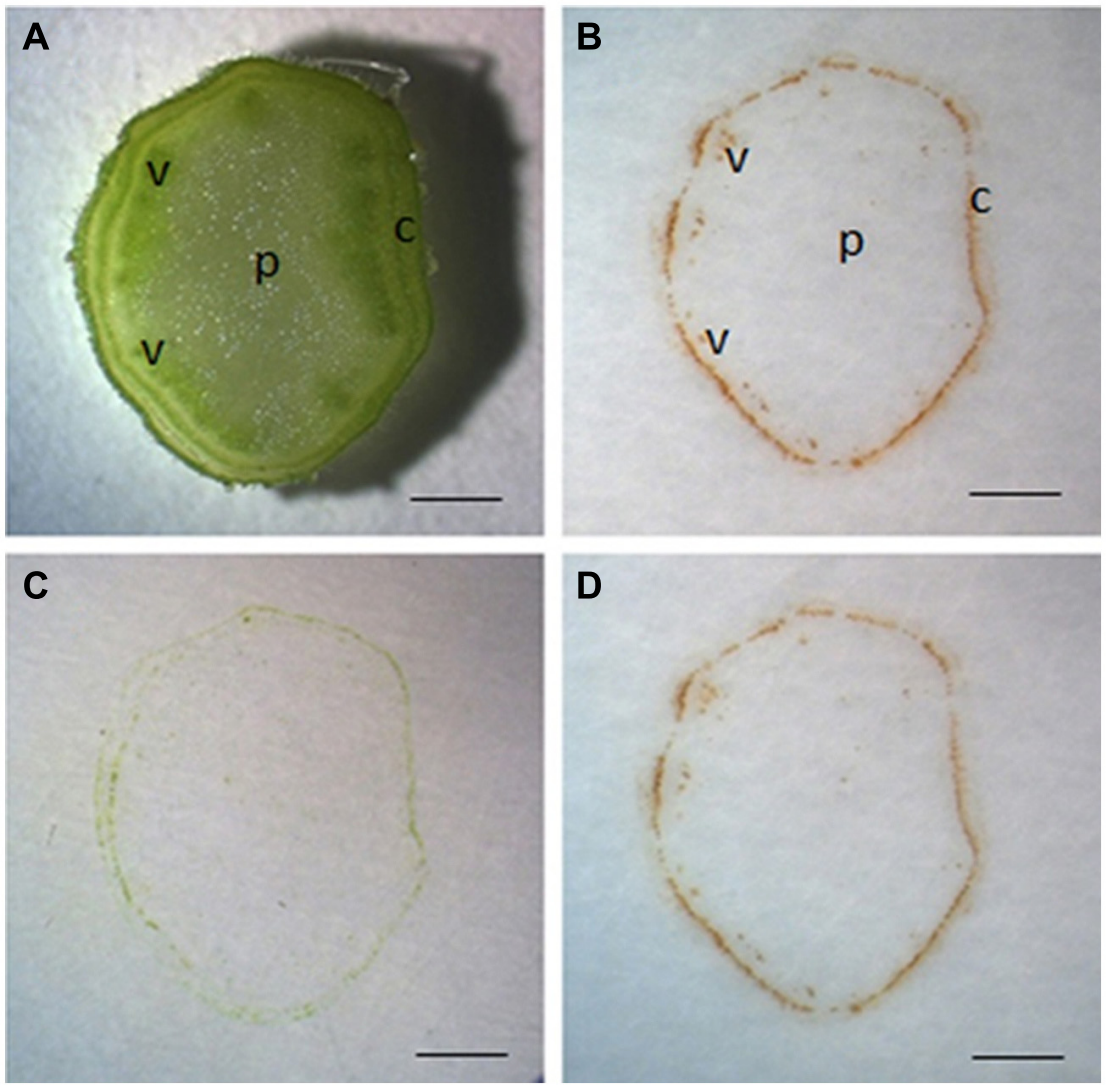
**Localization of H_2_O_2_ by DAB-mediated tissue printing in stems of tomato seedlings.** Free-hand sections (1 mm thick) were cut from the middle of tomato stem for tissue printing **(A)**. The sections were pressed for 10 s on nitrocellulose membrane impregnated in 5 mg mL^-1^ DAB–HCl solution (pH3.8) and photographed immediately **(B)** or 1 h later **(D)**. To verify the specificity of reaction, the sections were also pressed to membrane impregnated in 5 mg mL^-1^ DAB–HCl solution (pH3.8) plus 100 mM ascorbic acid **(C)**. Sections used in this figure were consecutive sections from the same stem. c, cortex; p, pith; v, vascular bundle. Scale bar = 1 mm in **(A–D)**.

Unless otherwise specified, tissue printing of tomato stem was always conducted on sections from the middle part of the seedling. We found that tissue printing of stem sections on nitrocellulose membrane for 10 s produced significant reddish-brown color at the cortex and vascular bundle regions (**Figures 1A,B**). To verify if the color is H_2_O_2_-specific, the reaction was done in the presence of ascorbic acid, a specific H_2_O_2_ scavenger (Thordal-Christensen et al., 1997). Since the nitrocellulose membrane needed to be air-dried for 30 min before it being used for tissue printing, it is possible that the soaked ascorbic acid might be oxidized. Therefore, to ensure there was sufficient reduced form ascorbic acid in membrane for the specific reaction with H_2_O_2_, we employed higher concentration of ascorbic acid (100 mM) than the commonly used concentration (10 mM; Thordal-Christensen et al., 1997; Salzer et al., 1999). It was found that no color was produced under ascorbic acid treatment (**Figure 1C**), indicating that the reaction was H_2_O_2_-specific. The same printing in **Figure 1A** was photographed again 1 h later with no significant changes in the H_2_O_2_ signal strength (**Figure 1D**). Furthermore, no obvious background color was developed following 1 h at room temperature (**Figure 1D**). Therefore, it can be concluded that our protocol to localize H_2_O_2_ in stem with DAB-mediated tissue printing is simple, rapid, and reliable.

### WOUND-INDUCED H_2_O_2_ IS AVOIDED IN THE REACTION

**FIGURE 2:**
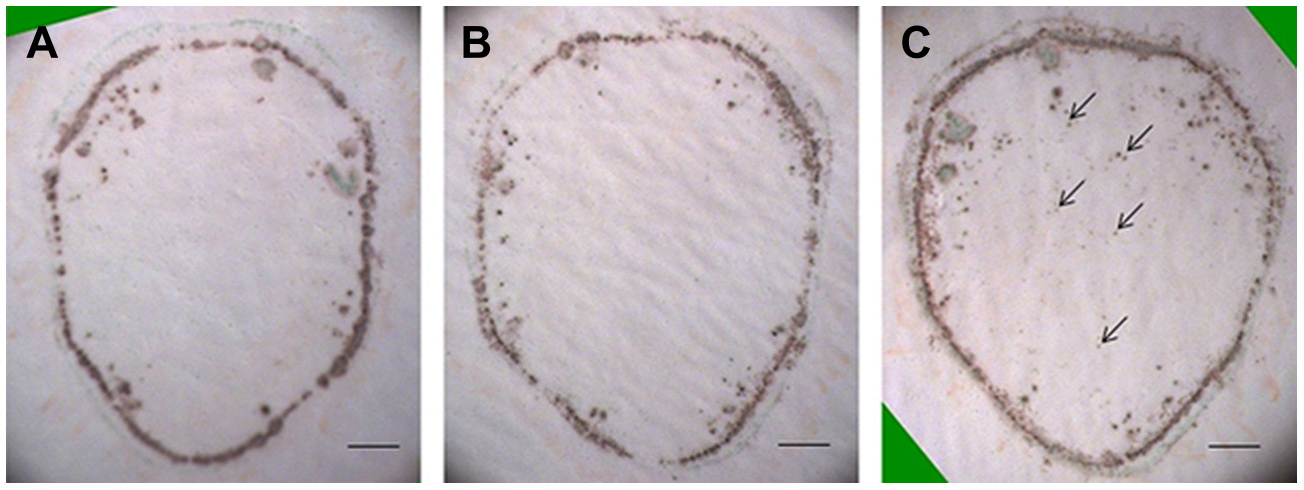
**Accumulation of wound-induced H_2_O_2_ in tomato stem after sectioning.** Free-hand sections (1 mm thick) were cut from the middle of tomato stem for tissue printing at 0 **(A)**, 1 **(B)**, and 2 **(C)** min after cutting. In **(A,B)**, no difference was found in H_2_O_2_ distribution pattern. In **(C)**, however, H_2_O_2_ was found in pith area (arrows) where no H_2_O_2_ was detected in **(A,B)**, indicating the accumulation of wound-induced H_2_O_2_ at 2 min after cutting. Sections used in this figure were consecutive sections from the same stem. Scale bar = 1 mm in **(A–C)**.

Previous studies have shown that H_2_O_2_ can be rapidly induced by wounding (Olson and Varner, 1993; Miller et al., 2009). To test the possible involvement of wound-induced H_2_O_2_ in the current protocol, tissue printing was performed at 0, 1, and 2 min after sectioning using consecutive sections. It was found there was no difference in H_2_O_2_ distribution between 0 and 1 min after sectioning, and H_2_O_2_ was mainly confined in the cortex and vascular bundles with no signal found in the pith (**Figures 2A,B**). However, obvious H_2_O_2_ signal was produced in the pith when tissue printing was conducted 2 min after sectioning (**Figure 2C**), indicating the production of wound-induced H_2_O_2_. These results indicate that although wound-induced H_2_O_2_ accumulated very quickly (within 2 min after cutting), the rapidity of our protocol (within 15 s, see Materials and Methods for details) can completely avoid the interference of wound-induced H_2_O_2_.

### ENDOGENOUS PEROXIDASE IS SUFFICIENT TO SUPPORT THE REACTION

**FIGURE 3:**
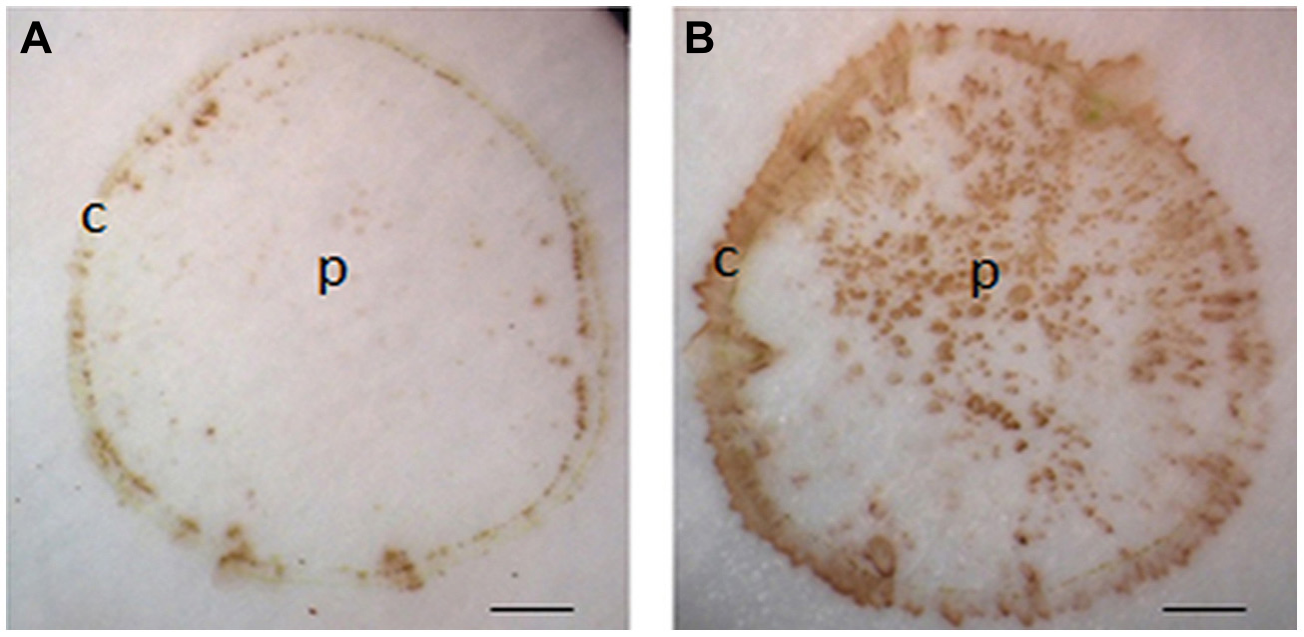
**Effects of exogenously applied H_2_O_2_ on H_2_O_2_ signal strength in tomato seedling stems.** Tissue printing was conducted on nitrocellulose membranes without **(A)** and with **(B)** exogenously supplied 20 mM H_2_O_2_. Note, application of exogenously supplied H_2_O_2_ increased the intensity of signals in the cortex, and with additional signals apparently detected in the pith region of tomato stem in **(B)** as compared to that in **(A)**. c, cortex; p, pith; Scale bar = 1 mm in **(A,B)**.

The reaction between DAB and H_2_O_2_ relies on the activity of peroxidase (Thordal-Christensen et al., 1997). In our protocol, H_2_O_2_ was localized using exogenous DAB and endogenous peroxidase. Undoubtedly, the exogenously supplied DAB used in our protocol was sufficient to support the reaction (Spruce et al., 1987; Thordal-Christensen et al., 1997). However, it is unknown whether endogenous peroxidase activity is enough for the reaction. To address this issue, exogenous H_2_O_2_ was introduced by soaking nitrocellulose in DAB solution containing 20 mM H_2_O_2_. If the activity of endogenous peroxidase from the stem section is more than enough for the reaction, the color intensity for H_2_O_2_ on tissue printing should become stronger in the presence of exogenously supplied H_2_O_2_. As expected, H_2_O_2_ was found in the cortex but not in the pith in the absence of exogenously supplied H_2_O_2_ (**Figure 3A**). However, H_2_O_2_ signal strength increased not only in the cortex but also appeared in the pith when the nitrocellulose membrane was pre-soaked with exogenous H_2_O_2_ (**Figure 3B**). These observations indicate that the activity of endogenous peroxidase was sufficient to support the reaction between endogenous H_2_O_2_ and exogenously supplied DAB.

### H_2_O_2_ DISTRIBUTION CHANGES DEVELOPMENTALLY IN TOMATO STEMS AND FRUITS

**FIGURE 4:**
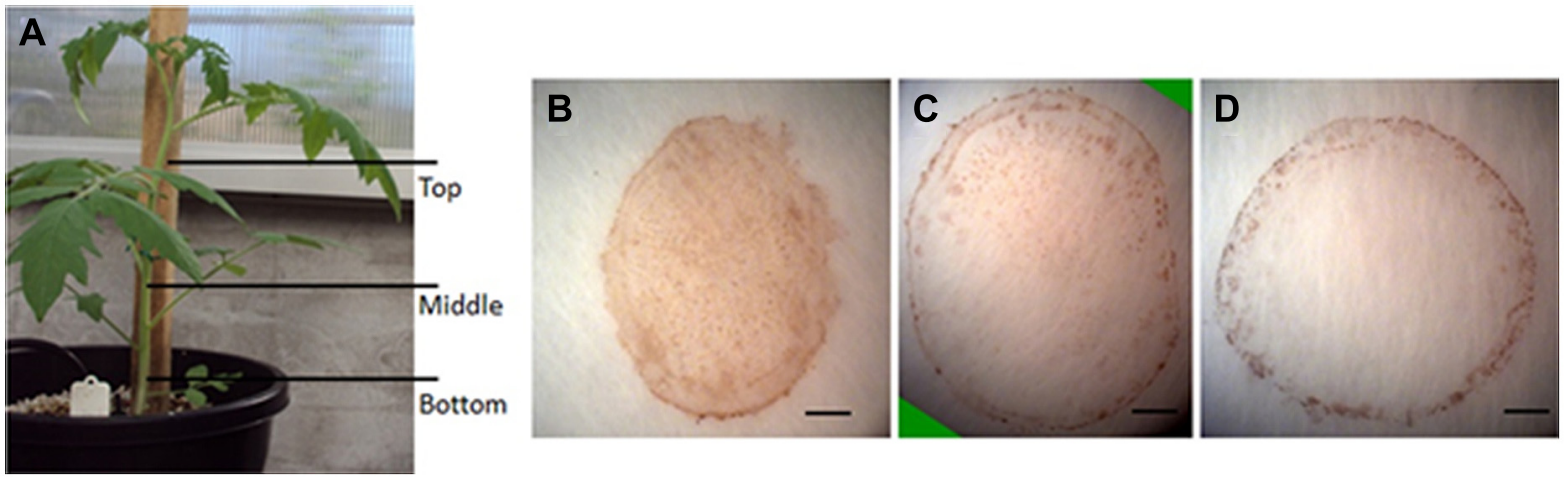
**Localization of H_2_O_2_ in different parts of tomato seedling stems.** Stem sections were cut from different positions as indicated in **(A)** for H_2_O_2_ localization at the top **(B)**, middle **(C)**, and bottom **(D)** of tomato stems using DAB-mediated tissue printing. Scale bars in **(B–D)** represent 1, 1.2, and 1 mm, respectively.

**FIGURE 5:**
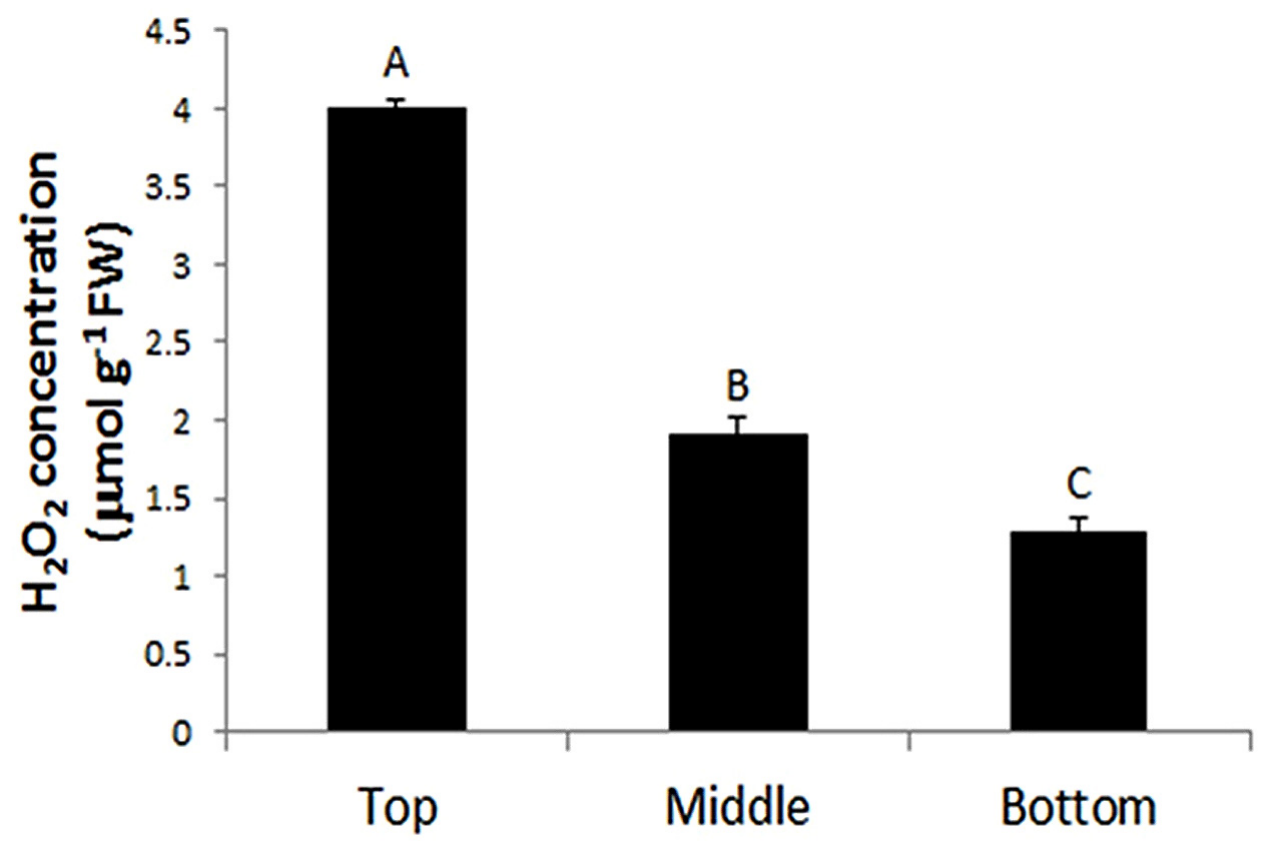
**Quantification of H_2_O_2_ at the top, middle, and bottom regions of tomato seedling stems.** Each value is the mean ± SE of four biological replicates (four stem samples from four individual seedlings). Values with different letters indicate significant differences (*P* ≤ 0.01).

The production of H_2_O_2_ is tightly regulated during plant development. For example, using starch/KI-mediated tissue printing, Schopfer (1994) showed that H_2_O_2_ level dramatically increases from the hook region toward the root in 5 days old soybean seedlings. Using the current protocol, we studied H_2_O_2_ distribution along the stem of tomato seedlings (**Figure 4A**). The analyzes revealed that the distribution patterns of H_2_O_2_ were strikingly different among the top, middle, and bottom regions of tomato seedling. H_2_O_2_ was distributed throughout the whole section at the top of seedlings (**Figure 4B**). However, the signal strength of H_2_O_2_ in pith decreased at the middle and bottom regions of the stem (**Figures 4C,D**), with H_2_O_2_ detected only in the cortex at the bottom area of the stem (**Figure 4D**). Accompanying the changed distribution pattern, H_2_O_2_ content appeared to decrease along stems from the top to the bottom. This observation on H_2_O_2_ gradient along tomato stems is contrary to that reported in soybean (Schopfer, 1994). The reason for the discrepancy may lie in different cultivation conditions. The soybean seedlings were grown in darkness (Schopfer, 1994), whereas our tomato seedlings were grown under normal conditions (10/14 h, day/night). To verify the reliability of our observation, H_2_O_2_ concentration was measured in tissue extracts at the top, middle, and bottom of tomato seedling stems. It was found that H_2_O_2_ content indeed decreased down the stem. H_2_O_2_ content at the top of seedling stem was one and two times higher than that at the middle and bottom part, respectively (**Figure 5**). The consistency between the quantified value (**Figure 5**) and the localized signal strength in sections (**Figure 4**) provides further evidence that our DAB-mediated tissue printing is reliable and can semi-quantitatively reflect H_2_O_2_ distribution in plant organs.

**FIGURE 6:**
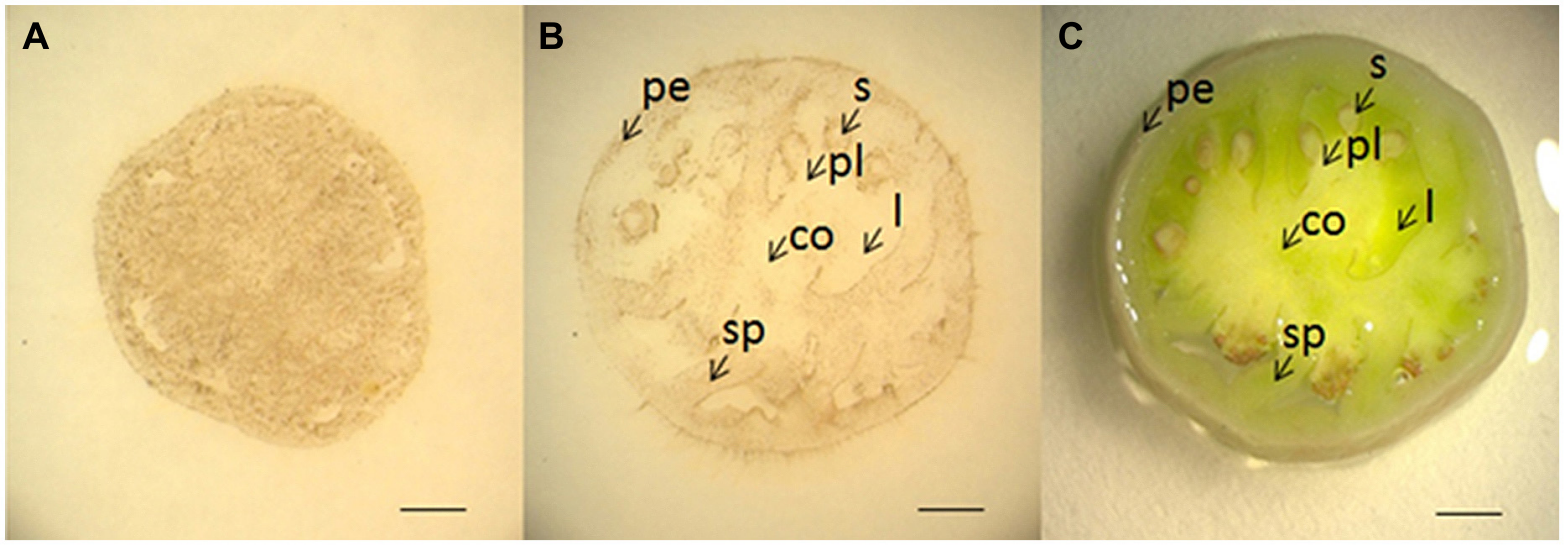
**Localization of H_2_O_2_ in young tomato fruits using DAB-mediated tissue printing.** Cross sections (1 mm thick) were cut transversely from the middle of tomato fruits at 5 **(A)** and 10 **(B)** DAF. **(C)** Shows the corresponding picture of fruit section used in **(B)**. co, columella; l, locule; pe, pericarp; pl, placenta; s, seed; sp, septum. Scale bars in **(A–C)** represent 1, 2, and 2 mm, respectively.

To test if the method is applicable to other large organs, we examined H_2_O_2_ localization in tomato fruits at 5 and 10 DAF using DAB-mediated tissue printing. At 5 DAF, H_2_O_2_ was detected abundantly and evenly throughout the section (**Figure 6A**). However, by 10 DAF, H_2_O_2_ appeared to be more tissue-specific, in which H_2_O_2_ was abundant in pericarp, seed, and septum, and no H_2_O_2_ signal was found in columella, locule, and placenta regions (Figures 6B,C). These results suggest that H_2_O_2_ distribution is also highly regulated during the development of tomato fruits.

It has been reported that ROS promotes cell division through accelerating auxin-mediated cell cycle entry in alfalfa (*Medicago sativa*; Fehér et al., 2008). ROS is also involved in the establishment and maintenance of root apical dominance in *Arabidopsis* (De Tullio et al., 2010) and formation of lateral roots in rice (Chen et al., 2013). Therefore, the higher level of H_2_O_2_ in the top of the stem (**Figures 4A** and **5**) and younger fruit (5 DAF; **Figure 6A**) might be indicative of high activities of cell division in these organs.

In conclusion, localization of H_2_O_2_ within large plant organs (e.g., stem and large fruit) other than thin and flat leaves is technically challenging. In this paper, we report the development of a DAB-mediated tissue printing method to localize H_2_O_2_ in tomato stem and fruit. The rapidity of our protocol (within 15 s) can effectively avoid the interference of wound-induced H_2_O_2_. This represents a major advantage over the protocol reported by Thordal-Christensen et al. (1997) where leaf stripes were incubated in DAB containing solutions for 8 h, inevitably leading to wound-induced H_2_O_2_ (Miller et al., 2009; Swanson et al., 2011). Another advantage is that the signal strength of H_2_O_2_ from our tissue printing was stably maintained for at least 1 h after tissue printing with no or little background developed. Furthermore, owing to high consistency between the signal intensity of localized H_2_O_2_ and its quantified concentration, the protocol can also be used to semi-quantitatively reflect the H_2_O_2_ distribution in plant organs. Thus, our protocol is a simple way to specifically, rapidly, and reliably localize H_2_O_2_ in large plant organs.

## Conflict of Interest Statement

The authors declare that the research was conducted in the absence of any commercial or financial relationships that could be construed as a potential conflict of interest.
